# Additions to the genus *Chroogomphus* (Boletales, Gomphidiaceae) from Pakistan

**DOI:** 10.3897/mycokeys.66.38659

**Published:** 2020-03-30

**Authors:** Munazza Kiran, Ammara Sattar, Khushbakht Zamir, Danny Haelewaters, Abdul Nasir Khalid

**Affiliations:** 1 Department of Botany, University of the Punjab, Quaid-e-Azam Campus, Lahore, 54590, Pakistan; 2 Department of Botany and Plant Pathology, Purdue University, USA; 3 Faculty of Science, University of South Bohemia, Czech Republic

**Keywords:** 2 new taxa, Basidiomycota, Boletales, coniferous forests, macrofungi, phylogeny, taxonomy

## Abstract

With only three published reports, the genus *Chroogomphus* (Boletales, Gomphidiaceae) is poorly studied in Pakistan. During recent sampling events in Khyber Pakhtunkhawa province, Pakistan, several collections of *Chroogomphus* were made, representing undescribed taxa. Based on morphological and molecular data, two new species are described: *Chroogomphus pakistanicus* and *C. pruinosus*. We present a description and illustrations for both taxa. A molecular phylogenetic reconstruction, based on the internal transcribed spacer (ITS1–5.8S–ITS2) barcode region, shows that *C. pakistanicus* and *C. pruinosus* are placed in two different subgenera of Chroogomphus (subg. Chroogomphus and subg. Siccigomphus, respectively).

## Introduction

*Chroogomphus* (Singer) Mill. was initially recognised as a sub-genus of *Gomphidius* Fr. (Singer 1948). It was Miller (1964) who elevated it to genus level. More than 33 taxa are currently recognised worldwide, including species, subspecies and varieties, but the number of accepted species in the genus is ambiguous (Miller and Aime 2001; Miller 2003; Watling 2004; Li et al. 2009; Martín et al. 2016; Razaq et al. 2016; Scambler et al. 2018). Members of the genus are characterised by ochraceous basidiomata; orange to somewhat ochraceous, decurrent lamellae; a fibrous veil; and grey to black spore deposit. Other useful characters are the pileipellis hyphae (moist to glutinous or viscid) and the stipe base (with hyphae that are amyloid in Melzer’s reagent) (Miller 1964; Miller and Aime 2001; Li et al. 2009; Martín et al. 2016).

The genus is currently divided into three subgenera – *Chroogomphus*, *Floccigomphus* (Imai) Niskanen, Scambler, & Liimat. and *Siccigomphus* Niskanen, Scambler, & Liimat. (Scambler et al. 2018). Subg. Chroogomphus includes species that have a pileipellis made of repent, gelatinised, narrow hyphae and a shiny pileus surface when dry (Miller and Aime 2001; Scambler et al. 2018). Members of subg. Floccigomphus are distinguished by a pileipellis composed of broad, filamentous, non-gelatinised hyphae, an unpolished pileus when dry and amyloid lamellar trama. Species of subg. Siccigomphus have inamyloid lamellar trama, smaller basidiospores and non-gelatinised pileipellis hyphae (Scambler et al. 2018).

*Chroogomphus* species are economically very important because of their ectomycorrhizal association with pines and applications as drugs and food (Agerer 1990, 1991; Miller 1964; Xie et al. 1986; Yu and Liu 2005; Dai and Tolgor 2007). They are found in Europe, America and Asia (Miller 1964; Miller and Aime 2001; Legon and Henrici 2005; Li et al. 2009; Knudsen and Taylor 2012; Scambler et al. 2018). In Pakistan, the genus is underexplored with only three published reports. These are *C. helveticus* (Singer) M.M. Moser, *C. roseolus* Yan C. Li & Zhu L. Yang and *C. rutilus* (Schaeff.) O.K. Mill. (Ahmad et al. 1997; Razaq et al. 2016). Here, we describe two new species of *Chroogomphus* belonging to two different subgenera, based on their morpho-anatomical features and molecular phylogenetic analysis.

## Materials and methods

### Sampling site

Specimens were collected from the Kumrat valley (35°32'N, 72°13'E, Siddiqui et al. 2013), district Upper Dir, Khyber Pakhtunkhwa, Pakistan. In this area, rainfall reaches 100–255 mm during monsoon season (Wahab 2011). The Panjkora River flows through the dense vegetation of the valley, which includes mixed pine forests. *Abies pindrow* Royle, *Cedrus deodara* (Roxb. ex D. Don) G. Don and *Pinus wallichiana* A.B. Jacks. are the main coniferous species (Shinwari et al. 2006).

### Morphological observations

Macro-morphological characters of fresh basidiomata were recorded and colour codes were assigned using Munsell Soil Color Charts (1975). Macro-morphological characters included the size, shape and colour of pileus; colour of gills and mode of attachment to the stipe; colour of stipe and attachment to the pileus; presence or absence of annular ring and volva. Micro-morphological features were observed using a compound light microscope (MX4300H, Meiji Techno, Japan). For detailed microscopic examination, sections of lamellae, pileipellis and stipitipellis from dried specimens were observed in 5% potassium hydroxide (KOH), Congo red stain and Melzer’s reagent. Anatomical features were measured using ScopeImage software version 1.0.0 (BioImager, Maple, Canada). Measurements of basidiospores were made under oil immersion. A minimum of 60 basidiospores, 20 basidia and 20 cystidia were measured. The abbreviations ‘n/m/p’ indicates number of basidiospores ‘n’, measured from ‘m’ basidiomata from ‘p’ collections. Basidiospores dimensions are given as length × width with extreme values given in parentheses; avQ = average Q of all spores ± standard deviation. Voucher specimens are deposited in LAH (Department of Botany, University of the Punjab, Pakistan).

### DNA extraction, PCR amplifications and sequencing

Genomic DNA was extracted from dried tissue employing a modified CTAB protocol (Gardes and Bruns 1993). Amplification of the internal transcribed spacer (ITS, including ITS1, 5.8S and ITS2) barcode region of the nuclear ribosomal DNA was done using the primer pair ITS1F and ITS4 (Gardes and Bruns 1993; White et al. 1990). Polymerase chain reaction (PCR) was performed in a reaction volume of 20 µl containing 10 µl of 2× PCR buffer (Sigma-Aldrich, St. Louis, Missouri), 0.1 µl of each 0.6 nM primer, 8.8 µl of ddH_2_O and 1 µl of template DNA under the following cycling parameters: initial denaturation at 94 °C for 1 min; followed by 35 cycles of denaturation at 94 °C for 1 min, annealing at 53 °C for 1 min and extension at 72 °C for 1 min; and a final extension at 72 °C for 8 min. Amplified PCR products were directly sequenced in both directions by Sanger sequencing, using the same primers (Macrogen Inc., South Korea). Consensus sequences were generated using BioEdit software version 7.2.5.0 (Hall 1999) and then blasted against the NCBI GenBank database (https://blast.ncbi.nlm.nih.gov/).

### Sequence alignment and phylogenetic analysis

We constructed an ITS dataset of our newly generated sequences along with closely related sequences that were downloaded from GenBank (Li et al. 2009; Martín et al. 2016; Scambler et al. 2018). We included species of *Gomphidius* Fr. as outgroup taxa (Scambler et al. 2018). Multiple sequence alignment was done using MUSCLE (Edgar 2004) available from EMBL-EBI (http://www.ebi.ac.uk/Tools/msa/muscle/). The final alignment was submitted to TreeBASE under study ID: S24298.

The ITS1, 5.8S and ITS2 loci were extracted from the aligned ITS dataset, allowing the selection of substitution models for each partition. Models were selected using ModelFinder (Kalyaanamoorthy et al. 2017) by considering the corrected Akaike Information Criterion (AICc). Selected models were TNe+G4 (ITS1, -lnL = 4480.541), K2P (5.8S, -lnL =754.828) and TIM3e+G4 (ITS2, -lnL = 4453.285). Phylogenetic relationships were inferred by Maximum Likelihood (ML) using IQ-TREE (Nguyen et al. 2015) from the command line, under partitioned models (Chernomor et al. 2016). Ultrafast bootstrapping was done with 1000 replicates (Hoang et al. 2017).

A Bayesian Inference (BI) phylogeny was estimated using BEAST version 1.8.4 (Drummond et al. 2012) with an uncorrelated lognormal relaxed clock, allowing for evolutionary rates to vary across branches. We selected a Birth-Death Incomplete Sampling speciation model (Stadler 2009) tree prior and appropriate substitution models as determined by jModelTest2 (Darriba et al. 2012) under AICc. Models were TrNef+G (ITS1, -lnL = 2028.8929), JC (5.8S, -lnL = 320.6928) and TPM3+G (ITS2, -lnL = 1905.6932). Four independent runs were performed from a random starting tree for 40 million generations with a sampling frequency of 4000. The analyses were run from the BEAST on XSEDE tool on the Cipres Science Gateway (Miller et al. 2010). Resulting log files were entered in Tracer (Rambaut et al. 2014) to check trace plots and burn-in values. Effective sample sizes were well over 200 for all sampled parameters for each run and so we selected a standard burn-in of 10%. After the removal of 10% of each run as burn-in, log files and trees files were combined in LogCombiner. TreeAnnotator was used to generate consensus trees (with 0% burn-in) and to infer the Maximum Clade Credibility tree.

Final phylogenetic reconstructions with ML bootstrap values (BS) and BI posterior probabilities (pp) were visualised in FigTree v1.4.3 (http://tree.bio.ed.ac.uk/software/figtree/) and edited in Adobe Illustrator version 23.0.6 (San Jose, California).

## Results

### Phylogenetic analyses

Amplification of the ITS from three basidiomata of *C. pruinosus* resulted in 670 bp sequences (GenBank accession numbers MK509768, MK509769 and MK509770). All of these sequences showed 97% similarity to *C. roseolus* (LT576117, Pakistan) with 100% query coverage. The ITS sequences obtained from two basidiomata of *C. pakistanicus* (MK509771, MK509772) were 650 bp in length and showed 98% similarity to *C. filiformis* Yan C. Li & Zhu L. Yang (EU706324, China) with 95% query coverage.

**Figure 1.**
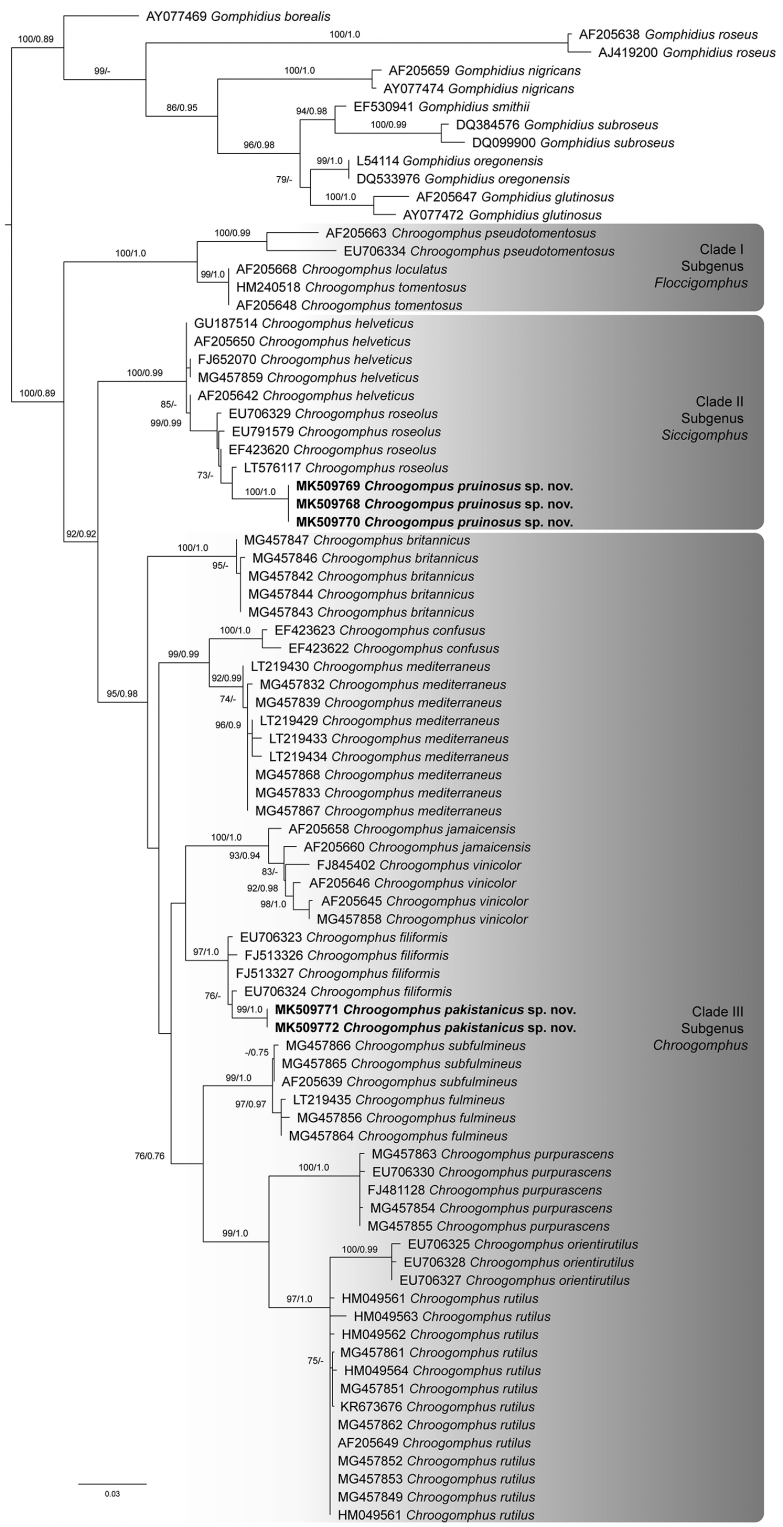
The best-scoring ML tree (-lnL = 4385.926) of the genus *Chroogomphus*, reconstructed from the ITS dataset. ML bootstraps (> 70%) and posterior probabilities (> 0.75) are indicated above or in front of the branch leading to each node. Newly described species are in boldface.

The ITS1–5.8S–ITS2 dataset included a total of 768 characters for 84 sequences including *Gomphidius* spp. as outgroup taxa (Suppl. material 1: Table S1). Out of 768 characters, 309 were of ITS1, 161 of 5.8S and 298 of ITS2; 121 (ITS1), 8 (5.8S) and 116 (ITS2) characters were parsimony-informative; and 164 (ITS1), 148 (5.8S) and 158 (ITS2) characters were constant. In the phylogenetic analysis of the ITS dataset (Figure 1), three main clades of *Chroogomphus* were recovered, representing the different subgenera: subg. Floccigomphus (clade I, maximum support), subg. Siccigomphus (clade II, BS = 100%, pp = 0.99) and subg. Chroogomphus (clade III, BS = 95%, pp = 0.98). The two isolates of *C. pakistanicus* sp. nov. formed a monophyletic clade (MLBS = 99%, pp = 1.0) within subg. Chroogomphus, sister to *C. filiformis*. Our three collections of *C. pruinosus* sp. nov. formed a separate clade with maximum support within subg. Siccigomphus, sister to *C. roseolus*.

### Taxonomy

#### Chroogomphus pakistanicus

Taxon classificationFungiBoletalesGomphidiaceae

M. Kiran & A.N. Khalid sp. nov.

0229D867-9B86-5791-9306-EC624FF8EE68

829715

Figures 2 , 3

##### Diagnosis.

Differs from *Chroogomphus filiformis* by the pileus ranging in colour from greyish-yellow brown to dark bluish-grey to orange and by the absence of a pinkish mycelium at the base of the stipe.

##### Types.

***Holotype***: Pakistan, Khyber Pakhtunkhwa province, district Dir (Upper), Kumrat valley, 35°32'N, 72°13'E, 2400 m a.s.l., gregarious on forest floor, 20 Aug 2016, *M. Kiran & A.N. Khalid*, KM82 (LAH35889), GenBank accession number MK509771 (ITS). ***Paratype***: *ibid.*, KM83 (LAH35890), GenBank accession number MK509772 (ITS).

##### Etymology.

Referring to the country where the type collections were collected.

##### Habitat.

On forest floor under mixed conifers.

##### Description.

*Basidiomata* small to medium-sized. *Pileus* 2–5 cm in diameter, secotioid when young, expanding broadly-parabolic to hemispherical towards maturity, radially fibrillose, ranging in colour from greyish-yellow brown (2.5Y,5/2) to dark bluish-grey (5BG,4/1) to orange (5YR,6/6), surface shiny or glistening, smooth, margin inrolled initially becoming straight to slightly seriate when mature. *Lamellae* adnate to slightly decurrent, distant, regular, concolorous to pileus, smooth, entire, lamellulae in two tiers, alternating with lamellae, short. *Stipe* 3–5.5 × 1 cm, central, more or less equal or sometimes enlarged at base, orange (5YR7/8) to reddish-brown (2.5YR4/8), pruinose to fibrillose to squamulose, with pinkish-white mycelium at stipe base, universal and partial veil absent. Odour and taste not recorded.

**Figure 2.**
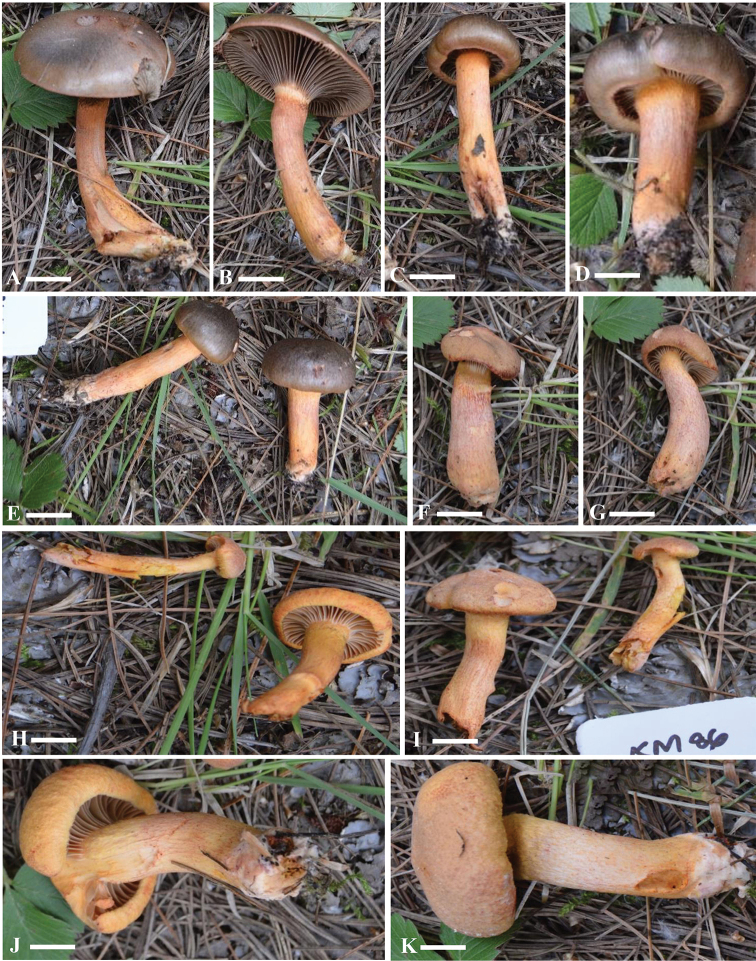
Basidiomata of *Chroogomphus* spp. **A–E***Chroogomphus pakistanicus***A, B**LAH35889, holotype **C–E**LAH35890**F–K***Chroogomphus pruinosus***F, G**LAH35887**H, I**LAH35888; **J, K**LAH35886, holotype. Scale bars: 1 cm.

*Basidiospores* [60/3/2], (15–)16–19.5(–20.5) × (5.5–)6–7.5(–8) µm, avl × avw = 17.5 × 6.6 µm, Q = (2.1–)2.2–3(–3.5) µm, avQ = 2.56±0.33 µm, oblong to elongate, mono-guttulate to multi-guttulate, pale brown in KOH, apiculus prominent, smooth, dextrinoid. *Basidia* 30–50 × 8–10.5 µm, avl × avw = 40 × 9 µm, hyaline to pale yellow in KOH, clavate to club–shaped. *Lamellar trama* yellowish hyphae in KOH, 5–11 µm, with brownish encrustations, inamyloid and non-dextrenoid. *Pleurocystidia* 75–107 × 17.5–25.5 µm, avl × avw = 91 × 43 µm, clavate to sometimes slightly utriform, pale brown to brown in KOH, encrusted, inamyloid. *Cheilocystidia* similar to pleurocystidia. *Pileipellis* a cutis, pale yellow to brownish KOH, 4–6 µm wide, amyloid, septate, clamped. *Pileal trama* composed of amyloid encrusted hyphae, 4–18 µm, yellowish in KOH. *Stipitipellis* a cutis of 3–9.5 µm wide, pale yellow to pale brown KOH, cylindrical, parallel, septate amyloid hyphae present at the base. Clamp connection present in all tissues.

##### Notes.

*Chroogomphus pakistanicus* can be easily distinguished from the other members in the genus by the unique bluish-grey colour of its pileus. The phylogenetically closest relative, *C. filiformis*, (Figure 1) is discriminated from *C. pakistanicus* based on the following morphological features: (1) the pileus of *C. pakistanicus* ranges in colour from greyish-yellow brown to dark bluish-grey to orange and has a glistening surface, whereas in *C. filiformis* the pileus is clearly olive grey to pinkish-orange; and (2) the pinkish mycelium at the base of the stipe typical for *C. filiformis* (Li et al. 2009) is absent in *C. pakistanicus*. *Chroogomphus britannicus* was included in sect. Filiformes by Scambler et al. (2018). In our phylogenetic tree, its position is unresolved within subg. Chroogomphus. Morphologically, it can be easily distinguished from the new species. *Chroogomphus britannicus* has larger basidiospores (20.3 × 7.1 µm), amyloid lamellar trama and inamyloid pileal trama (Scambler et al. 2018). The morphology of *Chroogomphus pakistanicus* is similar to *C. mediterraneus*, which can be distinguished by a subconical to convex pileus ranging in colour from grey to olivaceous to brown to red to pink to purplish, in combination with differently shaped cystidia, ranging from cylindrical, subfusiform, subutriform to sometimes subcapitate (Scambler et al. 2018). *Chroogomphus vinicolor* is another species related to *C. pakistanicus*, but the cystidia of *C. vinicolor* are thick-walled (5–7.5 µm) and it has a differently coloured pileus (Miller 1964; Singer and Kuthan 1976). Furthermore, geographically, members of the section Vinicolores have thus far only been reported from North America (Scambler at al. 2018). *Chroogomphus jamaicensis* may also be confused with *C. pakistanicus*, but it can be separated from the latter in having different micromorphological characters including thick-walled (4–5 µm) fusiform caulocystidia, which are occasionally amyloid towards the base (Miller 1964).

**Figure 3.**
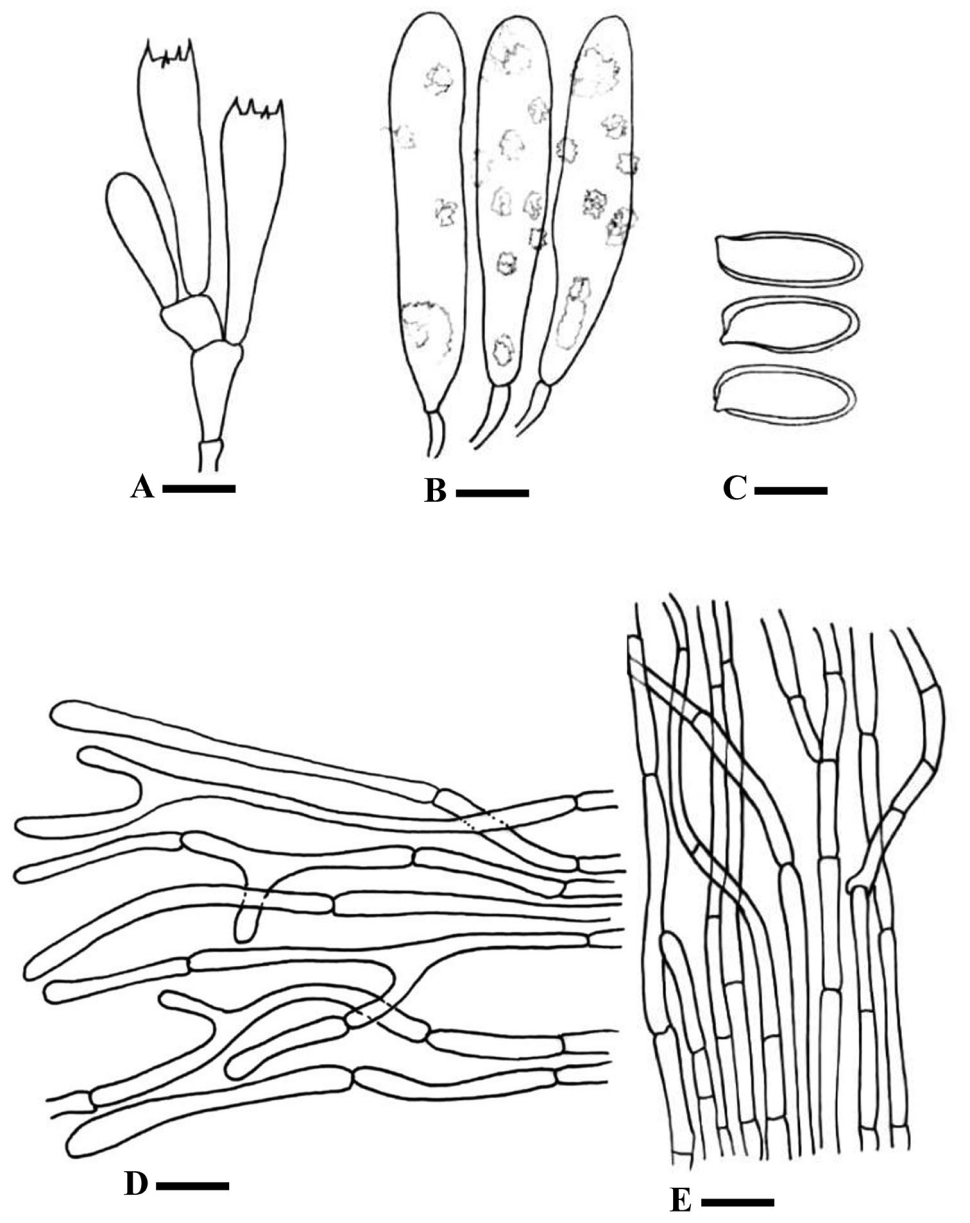
Line drawings of *Chroogomphus pakistanicus*. **A** Basidia **B** Cystidia **C** Basidiospores **D** Pileipellis hyphae **E** Stipitipellis hyphae. Scale bars: 12 µm (**A**), 17.5 µm (**B**), 8.5 µm (**C**), 25 µm (**D**), 30 µm (**E**).

#### Chroogomphus pruinosus

Taxon classificationFungiBoletalesGomphidiaceae

M. Kiran & A.N. Khalid sp. nov.

8DD2238E-DCB3-5CA5-A51D-36044068FB4C

829714

Figures 2 , 4

##### Diagnosis.

Differs from *Chroogomphus roseolus* by the pileal trama that is inamyloid in Melzer’s reagent and by the presence of pileocystidia and caulocystidia.

##### Types.

***Holotype***: Pakistan, Khyber Pakhtunkhwa province, district Upper Dir, Kumrat valley, 35°32'N, 72°13'E, 2400 m a.s.l., solitary or sub-gregarious on moisture rich loamy soil, 20 Aug. 2016, *M. Kiran & A.N. Khalid*, KM86 (LAH35886), GenBank accession MK509768 (ITS). ***Paratypes***: *ibid.*, KM85 (LAH35888), GenBank accession number MK509769 (ITS); *ibid.*, FS12 (LAH35887), GenBank accession number MK509770 (ITS).

##### Etymology.

Referring to the pruinose surface of pileus and stipe.

##### Habitat.

On forest floor under mixed conifers.

##### Description.

*Basidiomata* small to medium-sized, *Pileus* 0.5–3.5 cm in diameter, hemispherical, obtusely conic when young, expanding convex to broadly convex with maturity, margin inrolled initially becoming decurved, surface rough, pruinose, yellowish-orange to reddish-brown (7.5YR8/8–2.5YR4/8). *Lamellae* decurrent, sub-distant to distant, regular, broad up to 0.5 cm, forked near margin, light yellowish-orange (10YR,8/3), gill margins even, smooth, lamellulae in 2 tiers, alternating with lamellae. *Stipe* up to 4 cm long, central, pruinose, yellowish-orange to reddish-brown (7.5YR8/8–2.5YR4/8) in colour, rough, with tawny basal mycelium, more or less equal to broader towards base, universal and partial veil absent. Odour and taste not recorded.

*Basidiospores* [60/3/3], (11–)15–19(–21) × (4–)4.5–8(–8.5) µm, avl × avw = 16.5 × 6.5 µm, Q = (2.2–)2.3–3.4(–3.5), avQ = 2.64±0.43 µm, pale yellow to pale grey-brown in KOH, elongate to somewhat ellipsoid, slightly thick–walled, apiculate, dextrinoid, mostly mono-guttulate, germ pore absent. *Basidia* 37–53 × 7–13 µm, avl × avw = 41 × 11 µm, hyaline in 5% KOH, clavate, clamped at base, four-spored. *Lamellar trama* made up of hyphae, 3–6 µm, yellowish in KOH, encrusted, hyphae inamyloid with no or slightly amyloid encrustations, non-dextrenoid. Pleurocystidia 87–112 × 15–23 µm, avl × avw = 93 × 18 µm, hyaline with pale yellow walls in KOH, abundant, encrusted. *Cheilocystidia* similar to pleurocystidia but slightly smaller. *Pileipellis* an ixocutis of radially arranged hyphae, 10–12 µm in diameter, yellow to pale brown in KOH, inamyloid, with thin encrusted walls, cylindrical, septate, clamped. *Pileocystidia* 47–65 × 15–22 µm (avl × avw = 55 × 20 µm), similar to hymenial cystidia, pale yellow to pale brown in KOH. *Pileal trama* composed of yellowish hyphae with brownish encrustation in KOH, 12–20 µm, inamyloid and non-dextrenoid. *Stipitipellis* 6–12 µm, pale brown in KOH, inamyloid, straight, cylindrical, smooth and parallel. *Caulocystidia* 37–111.5 × 7–13.6 µm (avl × avw = 76.5 × 10.25 µm), rare, similar to hymenial cystidia.

**Figure 4.**
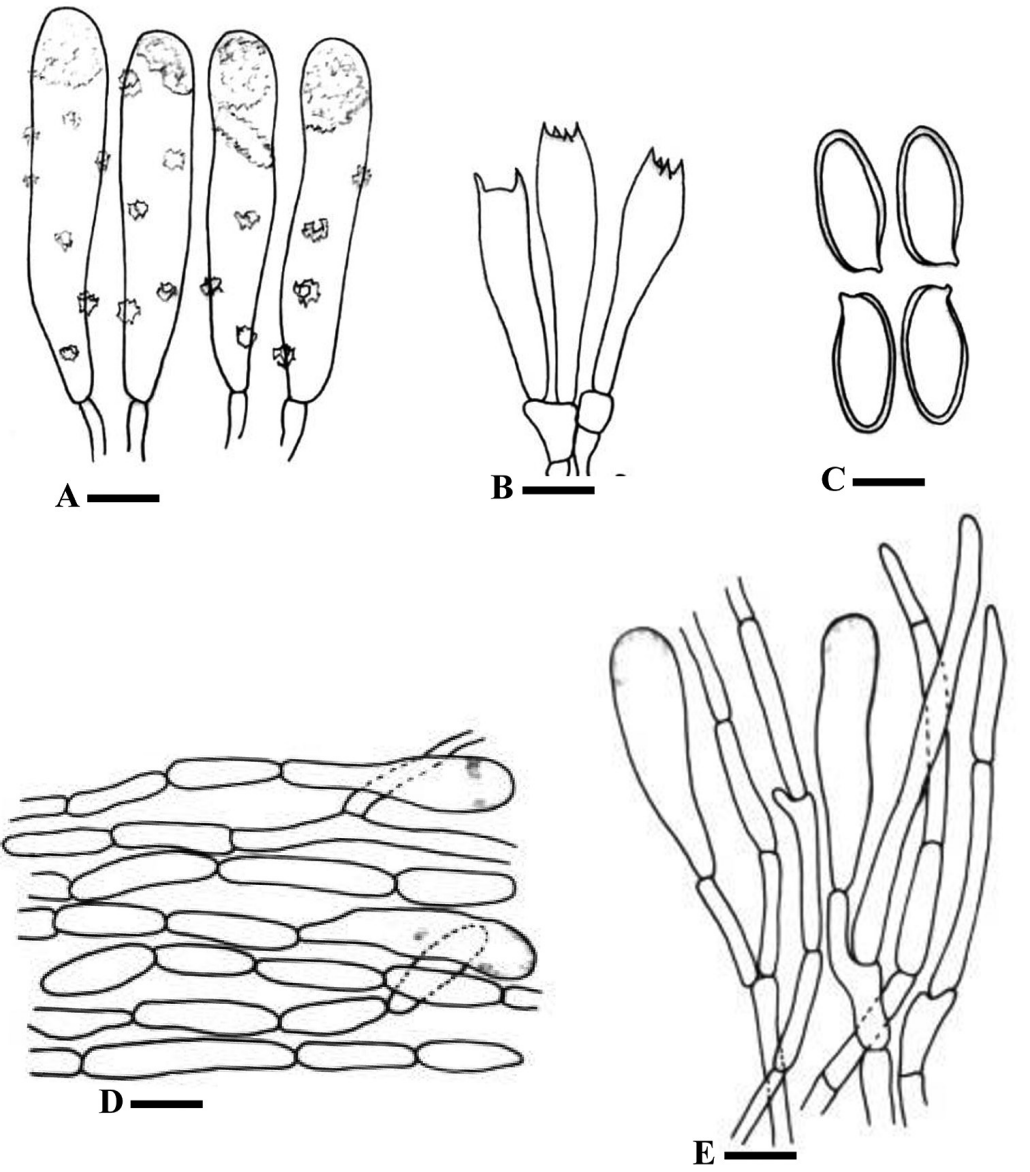
Line drawings of *Chroogomphus pruinosus*. **A** Basidia **B** Cystidia **C** Basidiospores **D** Pileipellis hyphae **E** Stipitipellis hyphae. Scale bars: 17.5 µm (**A**), 12 µm (**B**), 8 µm (**C**), 26.5 µm (**D**), 45 µm (**E**).

##### Notes.

*Chroogomphus pruinosus* differs from all other members of the genus in having pileocystidia. This new species is phylogenetically most closely related to *C. roseolus*, a species that has been reported from China and Pakistan (Li et al. 2009; Razaq et al. 2016). The macro- and micro-morphology of *C. pruinosus* is different from *C. roseolus* in the following characters: *C. pruinosus* possesses an obtusely conic to broadly convex, yellowish-orange, pruinose, larger pileus; presence of pileocystidia and caulocystidia in *C. pruinosus*; and the pileal and lamellar trama and stipitipellis of *C. pruinosus* are inamyloid, whereas those of *C. roseolus* are amyloid or partially amyloid (Li et al. 2009; Razaq et al. 2016). *Chroogomphus helveticus* is another close relative of *C. pruinosus* and has also been reported from China and Pakistan (Li et al. 2009; Razaq et al. 2016). However, no herbarium specimens are available for the Pakistani reports of *C. helveticus* (Ahmad et al. 1997) and it is likely that these collections represent *C. roseolus*, as discussed by Razaq et al. (2016). *Chroogomphus roseolus* is an Asian native species, whereas reports of *C. heleveticus* have so far only been confirmed in Europe, generally in association with 5-needle pines – mostly *Pinus cembra* (Li et al. 2009), which does not occur in Pakistan. A striking feature of *C. helveticus* is the presence of a pinkish mycelium at the base of the stipe (Li et al. 2009; Razaq et al. 2016; Scambler et al. 2018), which is not observed in *C. pruinosus*. *Chroogomphus rutilus* and *C. purpurascens* are morphologically very similar to *C. pruinosus*. However, *C. rutilus* has larger basidiomata (20–90 mm) with vinaceous brown or ochraceous-buff to vinaceous red, reddish-brown to purplish, umbonate pileus, buff to yellowish mycelium on the base of the stipe, slightly larger basidiospores (18.0 × 6.2 µm), cylindrical to subfusiform thick walled cystidia and lamellar trama composed of amyloid hyphae (Singer 1949; Miller 1964; Singer and Kuthan 1976; Gerhardt 1984; Breitenbach and Kränzlin 1991; Villarreal and Heykoop 1996; Horak 2005; Li et al. 2009; Scambler et al. 2018). *Chroogomphus purpurascens* is distinguished by a grey to brown then purple pileus that is slightly depressed, an ochraceous stipe, salmon to purple pink mycelium on the base of the stipe, thin-walled cystidia and deeply amyloid pileal trama. Moreover, the species is only known to be in association with *Pinus cembra*, *P. koraiensis* and *P. tabuliformis*, three pine species that are not found in Pakistan (Vassiljeva 1950, 1973; Azbukina 1990; Li et al. 2009). *Chroogomphus tomentosus*, a species that has been reported from Asia (Li et al. 2009), can be distinguished by its larger basidiospores [15–25 × 6–8(9) µm], thick-walled cystidia (2–4 µm) and strongly amyloid lamellar and pileal trama (Miller 1964).

### Key to species of *Chroogomphus* reported from Pakistan

**Table:** 

1	Pileipellis hyphae non-gelatinised	**2**
–	Pileipellis hyphae gelatinised	**3 Subgenus Chroogomphus**
2	Lamellar trama amyloid, cystidia thick-walled	**Subgenus Floccigomphus**
–	Lamellar trama inamyloid, cystidia thin-walled	**4 Subgenus Siccigomphus**
3	Pileus umbonate, ochraceous to vinaceous, Pileipellis hyphae inamyloid	***Chroogomphus rutilus***
–	Pileus broadly parabolic, bluish-grey to orange, Pileipellis hyphae amyloid	***Chroogomphus pakistanicus***
4	Pileal trama amyloid, pileocystidia and caulocystidia absent	***Chroogomphus roseolus***
–	Pileal trama inamyloid, pileocystidia and caulocystidia present	***Chroogomphus pruinosus***

## Discussion

Many taxa of fungi have recently been described using an integrative approach, combining morphology, DNA data and ecology (e.g. Aime 2004; Singh et al. 2015; Accioly et al. 2019; Jumbam et al. 2019; Sochorová et al. 2019). This was also shown to be a useful approach in the delimitation of species within *Chroogomphus* (Scambler et al. 2018). The genus can be found throughout the Northern Hemisphere with the exception of only one species, *C. papillatus*, which was reported from the Southern Hemisphere by Raithelhuber (1974). There is morphological and molecular evidence of intercontinental distribution for *C. purpurascens* and *C. rutilus*, which both occur in Europe and Asia (Miller and Aime 2001; Li et al. 2009; Martín et al. 2016; Scambler et al. 2018).

Our phylogenetic tree, obtained from ML and BI analyses (Figure 1), is in accordance with Scambler et al. (2018), with the division of the genus into the subgenera *Chroogomphus*, *Floccigomphus* and *Siccigomphus*. Subg. Chroogomphus was further subdivided by Scambler et al. (2018) into four sections – sect. Chroogomphus, sect. Confusi, sect. Filiformes and sect. Fulminei – and one informal clade, *Vinicolores*. Two identical sequences of *C. pakistanicus* are nested within subg. Chroogomphus sect. Filiformes and three identical sequences of *C. pruinosus* cluster within subg. Siccigomphus. In our phylogeny, sect. Filiformes is not monophyletic; the position of *C. britannicus* within subg. Chroogomphus is unresolved. The other sections are retrieved as monophyletic in our phylogeny with high support: sect. Chroogomphus (with *C. orientirutilus*, *C. purpurascens* and *C. rutilus*), sect. Confusi (*C. confusus* and *C. mediterraneus*), sect. Fulminei (*C. fulmineus* and *C. subfulmineus*) and the informal *Vinicolores* clade (*C. jamaicensis* and *C. vinicolor*).

The subgenera in our phylogenetic analyses are also supported morphologically. Members of clade II fall in subg. Siccigomphus found all over the Northern Hemisphere and are similar in having comparatively smaller basidiospores and inamyloid lamellar trama. They can be distinguished from the members of clade III, which belong to subg. Chroogomphus and have a narrow pileipellis and shiny pileus surface and distributed throughout Eurasia, but not in North America. Clade I represents subg. Floccigomphus, with members that are found in North America and Asia, but not in Europe and recognised by non-gelatinised pileipellis hyphae and amyloid lamellar trama.

Based on the distinct and well-supported molecular phylogenetic placement of our Pakistani collections in combination with morphological differences with their closest described relatives, we confirm that they represent two new species in the genus *Chroogomphus*.

## Supplementary Material

XML Treatment for Chroogomphus pakistanicus

XML Treatment for Chroogomphus pruinosus

## Supplementary materials

Supplementary material 1 Table S1. Taxa used in molecular phylogenetic analysis with voucher, country, and ITS GenBank accession number Data type GenBank accession numbers and associated metadata https://binary.pensoft.net/file/395273This dataset is made available under the Open Database License (http://opendatacommons.org/licenses/odbl/1.0/). The Open Database License (ODbL) is a license agreement intended to allow users to freely share, modify, and use this Dataset while maintaining this same freedom for others, provided that the original source and author(s) are credited.Munazza Kiran, Ammara Sattar, Khushbakht Zamir, Danny Haelewaters, Abdul Nasir Khalid
