# Supplementary material for: Additions to the genus Chroogomphus (Boletales, Gomphidiaceae) from Pakistan
Source: MycoKeys. 2020 Mar 30;66:23–38. doi: 10.3897/mycokeys.66.38659 (PMC7136303; doi:10.3897/mycokeys.66.38659)
Supplement: Supplementary material 1 — Table S1. Taxa used in molecular phylogenetic analysis with voucher, country, and ITS GenBank accession number [file mycokeys-66-023-s001.docx]

**Table S1.** Taxa used in molecular phylogenetic analysis with voucher, country, and ITS GenBank accession number.

| Species | ITS | Voucher | Country | Source |
| --- | --- | --- | --- | --- |
| *Chroogomphus britannicus* | MG457844 | H:6059351 | Finland | Scambler et al. 2018 |
| *Chroogomphus britannicus* | MG457843 | K(M):233759 | Germany | Scambler et al. 2018 |
| *Chroogomphus britannicus* | MG457842 | H:6001678 | Finland | Scambler et al. 2018 |
| *Chroogomphus britannicus* | MG457847 | H:6045578 | Finland | Scambler et al. 2018 |
| *Chroogomphus britannicus* | MG457846 | H:6025417 | Finland | Scambler et al. 2018 |
| *Chroogomphus confusus* | EF423623 | HKAS50407 | China | Li et al. 2009 |
| *Chroogomphus confusus* | EF423622 | HKAS50537 | China | Li et al. 2009 |
| *Chroogomphus filiformis* | EU706323 | KUN-HKAS 52934 | China | Li et al. 2009 |
| *Chroogomphus filiformis* | EU706324 | KUN-HKAS 52935 | China | Li et al. 2009 |
| *Chroogomphus filiformis* | FJ513327 | HKAS 54945 | China | Li et al. 2009 |
| *Chroogomphus filiformis* | FJ513326 | HKAS 54927 | China | Li et al. 2009 |
| *Chroogomphus fulmineus* | MG457856 | K(M):190394 | France | Scambler et al. 2018 |
| *Chroogomphus fulmineus* | MG457864 | LIP:0401321 | France | Scambler et al. 2018 |
| *Chroogomphus fulmineus* | LT219435 | JLS 3624 | Spain | Martin et al. 2016 |
| *Chroogomphus fulmineus* | AF205639 | ---- | Finland | Miller and Aime 2001 |
| *Chroogomphus helveticus* | AF205642 | VPI-OKM 21376 | Klausoden, Austria | Miller and Aime 2001 |
| *Chroogomphus helveticus* | GU187514 | taxon:132909 | Germany | Binder et al. 2010 |
| *Chroogomphus helveticus* | MG457859 | H:7019100 | Slovakia | Scambler et al. 2018 |
| *Chroogomphus helveticus* | FJ652070 | HKAS55293 | Czech Republic | Li et al. 2009 |
| *Chroogomphus helveticus* | AF205650 | ---- | Switzerland | Miller and Aime 2001 |
| *Chroogomphus jamaicensis* | AF205658 | ---- | Dominican Republic | Miller and Aime 2001 |
| *Chroogomphus jamaicensis* | AF205660 | ---- | Jamaica | Miller and Aime 2001 |
| *Chroogomphus mediterraneus* | LT219430 | JLS 3539 | Spain | Martin et al. 2016 |
| *Chroogomphus mediterraneus* | LT219429 | JLS 2917 | Spain | Martin et al. 2016 |
| *Chroogomphus mediterraneus* | LT219433 | JLS 3384 | Spain | Martin et al. 2016 |
| *Chroogomphus mediterraneus* | LT219434 | JCS 840B | Spain | Martin et al. 2016 |
| *Chroogomphus mediterraneus* | MG457832 | K(M):175418 | UK:Scotland | Scambler et al. 2018 |
| *Chroogomphus mediterraneus* | MG457839 | LIP:0401328 | France | Scambler et al. 2018 |
| *Chroogomphus mediterraneus* | MG457868 | FR2015401 | Greece | Scambler et al. 2018 |
| *Chroogomphus mediterraneus* | MG457833 | K(M):233761 | Germany | Scambler et al. 2018 |
| *Chroogomphus mediterraneus* | MG457867 | ML411181/1 | Cyprus | Scambler et al. 2018 |
| *Chroogomphus orientirutilus* | EU706325 | KUN-HKAS 52932 | China | Li et al. 2009 |
| *Chroogomphus orientirutilus* | EU706327 | KUN-HKAS 51180 | China | Li et al. 2009 |
| *Chroogomphus orientirutilus* | EU706328 | KUN-HKAS 52671 | China | Li et al. 2009 |
| *Chroogomphus pakistanicus* | **MK509771** | **LAH35889** | **Pakistan** | **This paper** |
| *Chroogomphus pakistanicus* | **MK509772** | **LAH35890** | **Pakistan** | **This paper** |
| *Chroogomphus pruinosus* | **MK509768** | **LAH35886** | **Pakistan** | **This paper** |
| *Chroogomphus pruinosus* | **MK509769** | **LAH35887** | **Pakistan** | **This paper** |
| *Chroogomphus pruinosus* | **MK509770** | **LAH35888** | **Pakistan** | **This paper** |
| *Chroogomphus pseudotomentosus* | AF205663 | ---- | ---- | Miller and Aime 2001 |
| *Chroogomphus pseudotomentosus* | EU706334 | KUN-HKAS 48726 | China | Li et al. 2009 |
| *Chroogomphus purpurascens* | EU706330 | HMJAU 3489 | China | Li et al. 2009 |
| *Chroogomphus purpurascens* | MG457863 | SOMF:29762 | Bulgaria | Li et al. 2009 |
| *Chroogomphus purpurascens* | MG457854 | K(M):233762 | UK | Scambler et al. 2018 |
| *Chroogomphus purpurascens* | MG457855 | H:6016159 | Finland | Scambler et al. 2018 |
| *Chroogomphus purpurascens* | FJ481128 | HKAS 54925 | Germany | Li et al. 2009 |
| *Chroogomphus roseolus* | LT576117 | PK-56 | Pakistan | Razaq et al. 2016 |
| *Chroogomphus roseolus* | EU706329 | KUN-HKAS 52901 | China | Li et al. 2009 |
| *Chroogomphus roseolus* | EF423620 | HKAS50552 | Yunnan, SW China | Li et al. 2009 |
| *Chroogomphus roseolus* | EU791579 | KUN-HKAS 52912 | China | Li et al. 2009 |
| *Chroogomphus rutilus* | HM049564 | taxon:85976 | China | Wang et al. 2011 |
| *Chroogomphus rutilus* | HM049563 | taxon:85976 | China | Wang et al. 2011 |
| *Chroogomphus rutilus* | HM049562 | taxon:85976 | China | Wang et al. 2011 |
| *Chroogomphus rutilus* | HM049561 | taxon:85976 | China | Wang et al. 2011 |
| *Chroogomphus rutilus* | MG457862 | LIP:0401324 | France | Scambler et al. 2018 |
| *Chroogomphus rutilus* | MG457851 | K(M):167792 | Italy | Scambler et al. 2018 |
| *Chroogomphus rutilus* | KR673676 | KA13-1203 | South Korea | Kim et al. 2015 |
| *Chroogomphus rutilus* | MG457861 | SOMF:29760 | Bulgaria | Scambler et al. 2018 |
| *Chroogomphus rutilus* | MG457849 | K(M):82320 | UK | Scambler et al. 2018 |
| *Chroogomphus rutilus* | AF205649 | ---- | Switzerland | Miller and Aime 2001 |
| *Chroogomphus rutilus* | MG457852 | K(M):198589 | Germany | Scambler et al. 2018 |
| *Chroogomphus rutilus* | MG457853 | K(M):175891 | Greece | Scambler et al. 2018 |
| *Chroogomphus subfulmineus* | MG457866 | LIP:0401318 | Cyprus | Scambler et al. 2018 |
| *Chroogomphus subfulmineus* | MG457865 | LIP:0401323 | Cyprus | Scambler et al. 2018 |
| *Chroogomphus tomentosus* | AF205668 | VPI-VTMH 3996 | Lane Co., OR, USA | Miller and Aime 2001 |
| *Chroogomphus tomentosus* | AF205648 | VPI-OKM 22998 | CA, USA | Miller and Aime 2001 |
| *Chroogomphus tomentosus* | HM240518 | UBC F19668 | Canada | Unpublished |
| *Chroogomphus vinicolor* | AF205645 | VPI-OKM 22514 | CA, USA | Miller and Aime 2001 |
| *Chroogomphus vinicolor* | AF205646 | taxon:5391 | ---- | Miller and Aime 2001 |
| *Chroogomphus vinicolor* | FJ845402 | SMI195 | Canada | Kranabetter et al. 2009 |
| *Chroogomphus vinicolor* | MG457858 | H:7031963 | USA | Scambler et al. 2018 |
| *Gomphidius borealis* | AY077469 | IBNR1999.0532 | Russia | Miller and Aime 2001 |
| *Gomphidius glutinosus* | AF205647 | ---- | USA | Miller and Aime 2001 |
| *Gomphidius glutinosus* | AY077472 | OKM27567 | USA | Miller and Aime 2001 |
| *Gomphidius nigricans* | AY077474 | OKM27830 | USA | Miller and Aime 2001 |
| *Gomphidius nigricans* | AF205659 | ---- | USA | Miller and Aime 2001 |
| *Gomphidius oregonensis* | DQ533976 | ---- | ---- | Unpublished |
| *Gomphidius oregonensis* | L54114 | TDB953b | USA | Kretzer et al. 1996 |
| *Gomphidius roseus* | AJ419200 | MA-Fungi 47696 | Spain | Martin and Raidl 2002 |
| *Gomphidius roseus* | AF205638 | ---- | Japan | Miller and Aime 2001 |
| *Gomphidius smithii* | EF530941 | UBC F16262 | Canada | Unpublished |
| *Gomphidius subroseus* | DQ099900 | OUC99229 | Canada | Durall et al. 2006 |
| *Gomphidius subroseus* | DQ384576 | UBC F15181 | Canada | Unpublished |

**References cited in Supplementary Table S1**

- Binder M, Larsson KH, Matheny PB, Hibbett DS. (2010) *Amylocorticiales* ord. nov. and *Jaapiales* ord. nov.: early diverging clades of Agaricomycetidae dominated by corticioid forms. Mycologia. 102(4):865-80.
- Durall DM, Gamiet S, Simard SW, Kudrna L, Sakakibara SM (2006) Effects of clearcut logging and tree species composition on the diversity and community composition of epigeous fruit bodies formed by ectomycorrhizal fungi. Botany. 84(6): 966–980.
- Kim CS, Jo JW, Kwag YN, Sung GH, Lee SG, Kim SY, Shin CH, Han, SK (2015) Mushroom flora of Ulleung-gun and a newly recorded *Bovista* species in the Republic of Korea. Mycobiology 43(3):239–257.
- Kranabetter JM, Friesen J, Gamiet S, Kroeger P (2009) Epigeous fruiting bodies of ectomycorrhizal fungi as indicators of soil fertility and associated nitrogen status of boreal forests. Mycorrhiza. 19(8): 535–548.
- Kretzer A, Li Y, Szaro T, Bruns TD (1996) Internal transcribed spacer sequences from 38 recognized species of *Suillus* sensu lato: phylogenetic and taxonomic implications. Mycologia. 88(5):776-85.
- Li YC, Yang ZL, Tolgor B (2009) Phylogenetic and biogeographic relationships of *Chroogomphus* species as inferred from molecular and morphological data. Fungal Diversity 38: 85–104.
- Martín MP, Siquier JL, Salom JC, Telleria MT, Finschow G (2016) Barcoding sequences clearly separate *Chroogomphus mediterraneus* (Gomphidiaceae, Boletales) from *C. rutilus*, and allied species. Mycoscience, 57(6): 384-392. https://doi.org/10.1016/j.myc.2016.06.004
- Miller OK, Aime MC (2001) Systematics, ecology and world distribution in the genus
  *Chroogomphus* (*Gomphidiaceae*). In: Misra JK, Horn BW (Eds.) *Trichomycetes* and other fungal groups. Science Publishers, Enfield, New Hampshire, 315–333.
- Scambler R, Niskanen T, Assyov B, Ainsworth AM, Bellanger JM, Loizides M, Moreau PA, Kirk PM, Liimatainen K (2018) Diversity of *Chroogomphus* (*Gomphidiaceae*, *Boletales*) in Europe, and typification of *C. rutilus*. IMA Fungus 9: 271–290. https://doi.org/10.5598/imafungus.2018.09.02.04
- Wang P, Liu Y, Yin Y, Jin H, Wang S, Xu F, Zhao S, Geng X (2011) Diversity of microorganisms isolated from the soil sample surround *Chroogomphus rutilus* in the Beijing region. International journal of biological sciences. 7(2):209.
